# Limited Vitrectomy versus Complete Vitrectomy for Epiretinal Membranes: A Comparative Multicenter Trial

**DOI:** 10.1155/2020/6871207

**Published:** 2020-10-20

**Authors:** Matteo Forlini, Purva Date, Domenico D'Eliseo, Paolo Rossini, Adriana Bratu, Andrea Volinia, Giovanni Neri, Tommaso Verdina, Maria Rosaria Carbotti, Gian Maria Cavallini, Luigi Sborgia, Alessandra Galeone, Aurelio Imburgia, Alessandro Mularoni, Alessandro Meduri

**Affiliations:** ^1^Division of Ophthalmology, San Marino Hospital, San Marino, CA, USA; ^2^Valvekar Medical & Research Centre, Solapur, Maharashtra, India; ^3^Department of Ophthalmology, “S. Maria delle Croci” Hospital, Ravenna, Italy; ^4^Institute of Ophthalmology, University of Modena and Reggio Emilia, Modena, Italy; ^5^Department of Ophthalmology, University of Bari, Bari, Italy; ^6^Clinic of Ophthalmology, Department of Biomedical Sciences, University of Messina, Messina, Italy

## Abstract

**Purpose:**

To evaluate whether limited vitrectomy is as effective as complete vitrectomy in eyes with epiretinal membrane (ERM) and to compare the surgical times and rates of complications.

**Methods:**

In this multicentre European study, data of eyes with ERM that underwent vitrectomy from January 2017 to July 2018 were analyzed retrospectively. In the limited vitrectomy group, a posterior vitreous detachment (PVD) was induced up till the equator as opposed to complete PVD induction till the vitreous base in the comparison group. Incidence of iatrogenic retinal breaks, retinal detachment, surgical time, and visual outcomes were compared between groups.

**Results:**

We included 139 eyes in the analysis with a mean age being 72.2 ± 6.9 years. In this, sixty-five eyes (47%) underwent limited vitrectomy and 74 eyes (53%) underwent complete vitrectomy. Iatrogenic retinal tears were seen in both groups (5% in limited vitrectomy versus 7% in complete vitrectomy, *p*=0.49). Retinal detachment occurred in 2 eyes in the limited vitrectomy group (3%) compared to none in the complete vitrectomy group (*p*=0.22). Best-corrected visual acuity (BCVA) and central macular thickness improved significantly with no intergroup differences (*p*=0.18). Surgical time was significantly shorter in the limited vitrectomy group with 91% surgeries taking less than 1 hour compared to 71% in the complete vitrectomy group (*p* < 0.001).

**Conclusion:**

A limited vitrectomy is a time-efficient and effective surgical procedure for removal of epiretinal membrane with no additional complications.

## 1. Introduction

The role of the posterior vitreous face has been established in the pathogenesis of many macular diseases such as macular hole, epiretinal membranes (ERM), and vitreomacular traction disorders [1, 2]. During surgical management of macular diseases, it is imperative to remove the posterior hyaloid face to gain access to the ERM and internal limiting membrane. Removal of the hyaloid also ensures relief from the anteroposterior tractional forces postulated to be causative in many of these diseases. Though the role of the postequatorial face of the posterior hyaloid is well established, the role of the peripheral hyaloid and vitreous cortex, including the firmly adherent vitreous base, is not fully understood in cases with macular pathologies.

Traditional vitreoretinal surgical teaching emphasizes that the entire vitreous must be removed while attempting any form of vitreoretinal surgery [3]. Though this may be true in cases of rhegmatogenous retinal detachment and other pathologies where the peripheral vitreous cortex is causative, this may not be true for eyes with predominantly macular pathologies. Performing a complete vitrectomy with base dissection can be time-consuming, requires panoramic wide-angled systems for proper visualization, is dependent on a skilled assistant for bringing the extreme peripheral cortex and ora serrata into view, and is associated with increased risk of lens touch and subsequent cataract formation in phakic eyes, especially in lesser-skilled surgeons and those with relatively lesser experience in these maneuvers. Since the peripheral vitreous cortex and vitreous base are challenging to excise thoroughly and are unlikely to be associated with most macular pathologies, it may be prudent not to attempt base excision and perform a limited vitrectomy alone in these cases [4].

However, leaving the preequatorial residual vitreous skirt may increase the risk of retinal tears and may predispose to a retinal detachment in these vitrectomised eyes. Bonfiglio et al. [5] have suggested limited vitrectomy for phakic eyes in cases with rhegmatogenous retinal detachment (RRD) without macular pathology with excellent results. Though minimal vitrectomy up to the equator was proposed almost a decade back by Boscia et al. [6], there have been no follow-up studies to establish the safety of this technique. We performed this multicentre study to primarily compare the efficacy of limited vitrectomy versus complete vitrectomy with base excision in eyes with ERM. The surgical time required and complication rates were secondary outcomes considered for analysis.

## 2. Methods

This retrospective study was carried out as per the tenets of the declaration of Helsinki. All patients had been well informed regarding the surgical procedure. Written informed consent was obtained from all patients. Data was obtained from the Institute of Ophthalmology, University of Modena and Reggio Emilia, Modena, Italy, Department of Ophthalmology, “S. Maria delle Croci” Hospital, Ravenna, Italy, and Department of Ophthalmology, University of Bari, Italy. The patient's identity was kept anonymous during data analysis and manuscript preparation. The study was carried out between January 2017 and July 2018. All patients with idiopathic ERMs who underwent standard 3-port pars plana vitrectomy (PPV) with or without combined phacoemulsification and intraocular lens implantation and having a minimum of 6-month follow-up were included in the data analysis. Eyes with other coexistent ocular pathologies such as corneal opacities, uveitis, and ERMs occurring secondary to other retinal pathologies such as trauma, previous retinal detachment, and retinal vascular disorders were excluded. Diabetics with any sign of retinopathy or maculopathy were excluded from the study.

All files of eligible patients were drawn from the electronic medical records from all the four participating centers using the ICD-9 coding system. The operating room registers were also used to identify eligible patients so that all consecutive patients operated for ERM during the study period were identified. Patient demographics, history of previous laser procedure, coexistent ocular pathology, baseline visual acuity, intraocular pressure, peripheral retinal degenerations, high-definition optical coherence tomography (HD-OCT) (Carl Zeiss Meditec, Inc., Dublin, CA)-based central macular thickness, presence of macular pucker, and the lens status were recorded from the case files. BCVA was measured by early treatment diabetic retinopathy study (ETDRS) charts and then converted into a logarithm of the minimum angle of resolution (logMAR) for statistical analysis.

**Figure 1:**
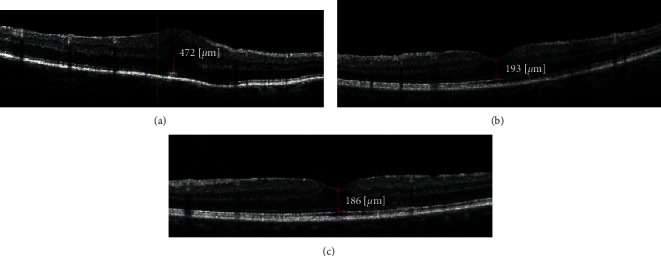
OCT scan of patient with limited vitrectomy with ERM removal. ((a)) Preoperative scan showing ERM with CMT of 472 *μ* with BCVA measuring 0.5 logMAR. ((b)) A one-month postoperative scan showing reduced CMT of 193 *μ* with BCVA improving to 0.8. ((c)) Yearly follow-up scan showing CMT 186 *μ* with normal foveal contour and vision improving to 0.9 logMAR.

**Figure 2:**
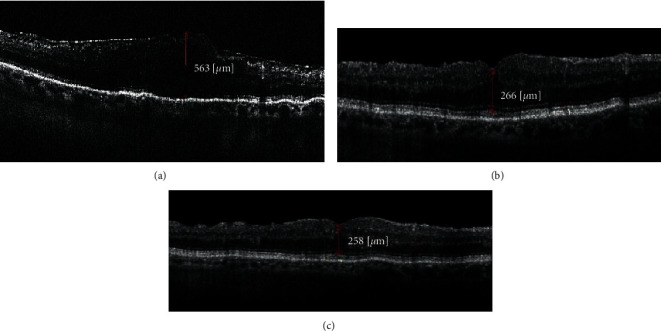
OCT scan of the patient with complete vitrectomy with ERM removal. ((a)) Preoperative scan showing ERM with loss of foveal contour, CMT measuring 563 *μ*, and BCVA measuring 0.5 logMAR. ((b)) The one-month postoperative scan showing a reduction in CMT to 266 *μ* with BCVA measuring 0.8. ((c)) After a year, CMT was 258 *μ* and BCVA improved to 0.9 logMAR.

Intraoperative parameters such as the gauge of instrumentation used, limited versus complete vitrectomy, peeling of ERM alone or combined with internal limiting membrane peeling (ILM), the stain used to delineate the ERM and ILM, and tamponade used at the end of surgery were noted. Intraoperative complications, especially the occurrence of peripheral retinal tears and the need for any laser photocoagulation, were also noted. The duration of surgery was recorded in 5 categories as 30–45 minutes, 46–60 minutes, 61–90 minutes, 91–120 minutes, and > 120 minutes. Data from follow-up visits at 1 week, 6 months, and the last follow-up was noted. It included BCVA, central macular thickness, and any complications, especially retinal detachment (Figures 1 and 2).

## 3. Surgical Procedure

**Figure 3:**
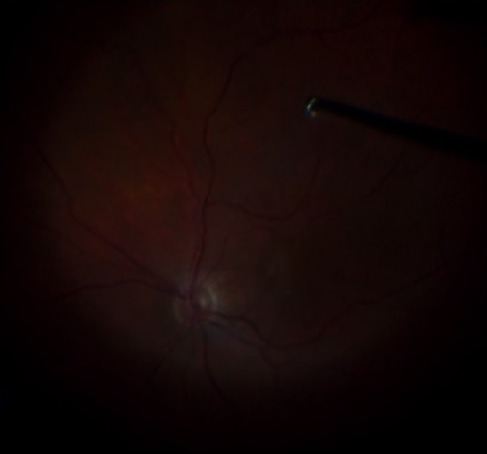
Limited vitrectomy: posterior vitreous detachment induction with “core vitrectomy.”

**Figure 4:**
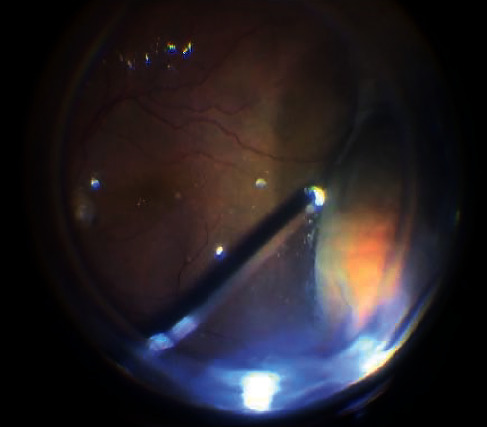
Complete vitrectomy: peripheral vitreous removal with vitreous base shaving using dynamic preequatorial scleral indentation.

**Figure 5:**
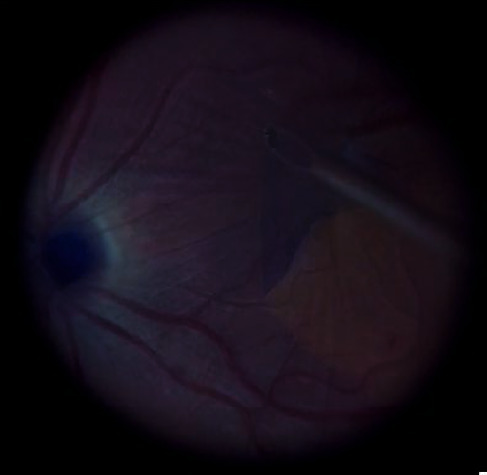
Internal limiting membrane peeling after brilliant blue staining after ERM removal.

The standard three-port pars plana vitrectomy (PPV) was carried out under local anesthesia. In eyes with coexistent cataract routine phacoemulsification via a clear corneal temporal incision with an intraocular lens (IOL), implantation was done. In eyes that underwent limited vitrectomy alone, after creating 3 standard ports at the pars plana, a PVD was induced up till the equator and the limited vitrectomy was completed without disturbing the peripheral cortical vitreous and vitreous base (Figure 3). In a complete vitrectomy, the PVD was propagated till the vitreous base and the entire vitreous body, including the peripheral cortex and base, was removed to the extent possible (Figure 4). In phakic eyes, under scleral indentation, the entire peripheral vitreous was visualized and removed. After vitrectomy, the ERM was stained with the preferred dye and peeled using microforceps. The ILM was then stained with a brilliant blue or dual dye and peeled around the center of the fovea for approximately 2 disc diameters (Figure 5). Ports were removed after using tamponade. The eye was filled with either saline or another tamponade as per the surgeons' choice. The gauge of vitrectomy, limited versus complete vitrectomy, type of stain used, and the type of tamponade were at the surgeon's discretion.

## 4. Statistical Analysis

All continuous variables were expressed as mean with standard deviation or median with interquartile range and group differences were analyzed using the Student *t*-test or Wilcoxon's rank-sum test for variables with the nonparametric distribution. Categorical variables were expressed as proportions (*n*%) and group differences were analyzed using the chi-square or Fisher's exact test. All data were entered in Microsoft Excel and analyzed using STATA 12.1 I/c (Fort Worth, Texas, USA), and *p* value of less than 0.05 was considered statistically significant.

## 5. Results

One hundred and thirty-nine eyes of 139 patients were included in the analysis. The mean age was 72.2 ± 6.9 years. Of these, 52% (73) were men and 66 were females. Diabetes was seen in 38 (27%) subjects and 63 (45%) were hypertensive on systemic medications. Sixty-five eyes (47%) underwent limited vitrectomy prior to ERM removal, whereas 74 eyes (53%) underwent complete vitrectomy with peripheral base excision to the extent possible. A comparison of baseline characteristics between these groups is shown in Table 1. Patients undergoing limited vitrectomy were marginally younger and more eyes in this group were phakic compared to those in the complete vitrectomy group. All other preoperative parameters including BCVA and central macular thickness were comparable between groups.

A comparison of the intraoperative characteristics between groups is shown in Table 2. About two-thirds of surgeries were performed using 25G instrumentation in the limited vitrectomy group compared to a significantly higher proportion of 23G in the complete vitrectomy group. A combination of ERM and ILM was peeled in significantly more eyes in the complete vitrectomy group. Similarly, a dual dye staining technique was used more often in the complete vitrectomy group while brilliant blue dye with negative ERM staining was more often used in the limited vitrectomy group. The air was the commonest tamponade used in the complete vitrectomy group compared to significantly greater use of nonexpansile gases and saline in the limited vitrectomy group. Iatrogenic peripheral retinal tears occurred in 8 eyes overall (6%) with no intergroup differences and all tears received prophylactic barrage laser intraoperatively. The surgical time was significantly reduced in the limited vitrectomy group with more than 90% of the surgeries completed in less than 1 hour compared to only 70% in the complete vitrectomy group.

After adjusting for possible confounders influencing the duration of surgery such as the operating surgeon, gauge of PPV used, lens status, and PVD status, we found that performing phacoemulsification along with PPV required 3.4 minutes more (95% confidence interval = 1.8 to 6.9 minutes) compared to PPV alone (*p*=0.04).  Table 3 shows a comparison of surgical time in both groups.

A comparison of outcomes and complications between the groups is shown in Table 4. The mean follow-up was 14.3 ± 2.3 months. At a 1-week follow-up, BCVA had improved to 0.5 ± 0.2 logMAR in the limited vitrectomy group and 0.43 ± 0.2 logMAR in the complete vitrectomy group (*p*=0.21). BCVA improved further in both groups compared to preoperative vision and there was no difference in vision between groups at 6 months. The central macular thickness was marginally lower in the limited vitrectomy group but this difference did not reach statistical significance. Retinal detachment was seen in two eyes (3%) in the limited vitrectomy group and in none of the eyes in the complete vitrectomy group. None of the retinal detachments occurred in eyes that had experienced iatrogenic retinal tears during surgery. One detachment occurred 10 months after surgery and the other occurred 2 months after surgery. Both underwent successful retinal reattachment surgery with silicone oil tamponade. Self-limiting cystoid macular edema was the commonest complication seen in fewer than 10% of the patients in both groups.

## 6. Discussion

In this multicentric retrospective European study, we found that performing a limited vitrectomy along with ERM peeling yielded equivalent results compared to a complete vitrectomy with base excision. Though both groups experienced the same number of iatrogenic retinal tears during surgery, there are chances of retinal detachment occurring in the limited vitrectomy group; however, the incidence was not statistically significant compared to the complete vitrectomy group. Limited vitrectomy was significantly faster with the majority of surgeries being completed in less than one hour.

Boscia et al. [6], in a noncomparative design, suggested limited vitrectomy for ERM and vitreomacular traction syndrome. They performed the same in 176 eyes using 25G vitrectomy systems. At a mean follow-up of 15 months, they found excellent visual and anatomical results and retinal detachment seen only in 2 eyes (1%). Similarly, Ozkaya et [4] performed limited vitrectomy with membrane peeling for ERM and IMH (idiopathic macular hole) in fifty-two eyes. They found a transient rise of intraocular pressure (IOP) in 3 (5.9%), endophthalmitis in 1 (2.0%), and retinal detachment in 1 patient (2.0%) during the follow-up.

In our comparative study, we found similar results in the cohort of eyes that underwent limited vitrectomy alone. Likewise, we also noticed retinal detachment in 2 eyes only. Spaide [7] has suggested a minimal vitrectomy by dissecting the connection between the vitreous and the retina. This was done under OCT guidance with a newly designed microspatula knife. The posterior vitreous was not stripped from the retinal surface. Limited vitrectomy over the hole was performed to create a space for a gas bubble. They reported successful results from 3 patients, without any significant complications. In another study, Kim et al. [8] performed posterior hyaloid separation slightly beyond the temporal vascular arcade in 59 eyes with macular disorders and compared it with 57 eyes that underwent complete vitrectomy. The incidence of iatrogenic peripheral retinal breaks was significantly lower in eyes with the partial vitrectomy (3.4%), compared to complete vitrectomy (16%). In our study, we observed peripheral breaks in 5% and 7% eyes undergoing limited and complete vitrectomy, respectively.

The main concern with performing a limited vitrectomy for macular pathologies is the possibility of condensation and contraction of the residual peripheral vitreous cortex. This may further lead to an increased risk of retinal tears with subsequent retinal detachment. The incidence of retinal detachment varies from 1 to 18% in previously reported studies [9–12]. In a large multicentric study from France, involving 474 eyes with macular pathologies, Matoni et al. [9] reported that iatrogenic retinal breaks were seen in 1.7% cases and an additional 2.7% experienced retinal detachment. In another large study on more than 1600 eyes using an ultrahigh-speed 25G cutter, Mura et al. [10] reported that the risk of iatrogenic breaks (1.8%, *n* = 25) is higher when a PVD is induced intraoperatively. Tarakcioglu et al. [13] postulated that induction and extension of PVD or performing peripheral vitreous shaving could be a cause of iatrogenic peripheral retinal tears. Another mechanism could be sclerotomy-associated breaks. Rahman et al. [12] reported a much higher incidence of iatrogenic retinal breaks (18%) in eyes with macular pathologies and attributed this to more adherent posterior hyaloid. Hence, it may be prudent to not induce a PVD beyond the equator while performing vitrectomy for macular pathologies. Thus, the vital step before concluding the surgery is to do a detailed examination of the periphery with indentation and prompt laser treatment whenever needed.

We found that performing a limited vitrectomy along with peeling the ERM and ILM was significantly faster than performing a complete vitrectomy in most instances. Reducing surgical time may improve surgical performance, especially in a high volume surgical setup. Additionally, the patient's subjective experience may also be better with a shorter surgical time. We did not find any other significant differences between eyes that underwent limited versus complete vitrectomy in terms of BCVA, macular thickness, or other postoperative complications such as cystoid macular edema.

The merits of this study are its multicentric nature and the presence of a comparison group. The drawbacks are the retrospective nature, relatively small sample size, and fewer cases of retinal tears and detachments making safety assessment difficult using statistical tools. So, we analyzed the rate of complications. Different surgeons and different settings could have had an influence on our results. The findings of our study may have limitations in cases of complex ERMs, where the surgical time may be prolonged.

## 7. Conclusion

In conclusion, we found that performing a limited vitrectomy is at least as effective as a complete vitrectomy in the management of macular pathologies. Limited vitrectomy also reduces the operative time, without increasing the rate of complications. Further prospective, randomized studies with larger sample sizes are required to confirm these observations.

## Figures and Tables

**Table 1:** Baseline demographic and clinical characteristics of eyes in limited versus complete vitrectomy group.

Variable	Limited vitrectomy (*n* = 65)	Complete vitrectomy (*n* = 74)	*p* value
Age (years)	71.03 ± 5.6	73.2 ± 7.8	0.06
Gender (% men)	35 (54%)	38 (51%)	0.77
Eye (% right eye)	38 (58%)	43 (58%)	0.96
BCVA (logMAR)	0.6 ± 0.1	0.5 ± 0.2	0.13
Central macular thickness (*μ*)	428 ± 93	454 ± 100	0.14
Previous h/o retinal laser	9 (14%)	8 (11%)	0.22
Lens status: clear lens	7 (11%)	6 (8%)	0.01
Cataractous lens	41 (63%)	29 (39%)	
Pseudophakia	17 (26%)	39 (53%)	
Coexistent glaucoma (%)	3 (5%)	6 (8%)	0.40

*Coexistent retinal pathology (%)*			
Peripheral degenerations	3 (5%)	0	0.34
Diabetic retinopathy	2 (3%)	1 (1.3%)	
High myopia	0	2 (2.7%)	
Polypoidal choroidal vasculopathy (PCV)	1 (1.3%)	0	

**Table 2:** Comparison of intraoperative characteristics in limited versus complete vitrectomy group.

Variable	Limited vitrectomy (*n* = 65)	Complete vitrectomy (*n* = 74)	*p* value
*Gauge used*			<0.001
23G	19 (29%)	29 (40%)	
25G	43 (66%)	32 (43%)	
27G	3 (5%)	13 (17%)	

*Peeling*			<0.001
ERM only	17 (26%)	2 (3%)	
ERM + ILM peeling	48 (74%)	72 (97%)	

*Stain used*			<0.001
Dual staining	9 (14%)	61 (82%)	
Brilliant blue dye	39 (60%)	3 (4%)	
Triamcinolone	11 (17%)	9 (12%)	
Others	6 (9%)	1 (1%)	
Phacoemulsification combined	39 (60%)	44 (59%)	0.95
Peripheral retinal breaks	3 (5%)	5 (7%)	0.49

*Tamponade used*			<0.001
Gas	9 (14%)	5 (7%)	
Air	43 (66%)	68 (92%)	
Saline	13 (20%)	1 (1%)	

**Table 3:** Comparison of duration of surgery of ERM + ILM peeling groups in limited versus complete vitrectomy groups.

Duration of surgery (mins)	Limited vitrectomy	Complete vitrectomy	Total	*p* value
30–45 min	2 (3.5%)	15 (20%)	17 (12%)	<0.0001
46–60 min	58 (89%)	37 (50%)	95 (68%)	
61–90 min	3 (4%)	17 (23%)	20 (15%)	
91–120 min	2 (3.5%)	4 (5.5%)	6 (4.3%)	
>120 min	0 (0%)	1 (1.5%)	1 (0.7%)	
Total	65 (100%)	74 (100%)	139 (100%)	

**Table 4:** Complications and outcomes of limited versus complete vitrectomy at the end of a 6-month follow-up.

Variable	Limited vitrectomy (*n* = 65)	Complete vitrectomy (*n* = 74)	*p* value
BCVA (logMAR)	0.3 ± 0.2	0.22 ± 0.2	0.18
Central macular thickness (*μ*)	286 ± 87	358 ± 75	0.09

*Complications*			
Retinal detachment	2 (3%)	0	0.22
Cystoid macular edema	5 (8%)	8 (11%)	0.45
Macular hole	1 (1.5%)	0	

## Data Availability

Data used to support the findings of this study are available from the corresponding author upon request.

## Supplementary Materials

Supplementary Materials Video 1: limited vitrectomy with ILM peeling. Video 2: total vitrectomy + ILM peeling with peripheral base shaving with endolaser.
